# Microbial biomass carbon and enzyme activities as influenced by tillage, crop rotation and residue management in a sweet sorghum cropping system in marginal soils of South Africa

**DOI:** 10.1016/j.heliyon.2020.e05513

**Published:** 2020-11-18

**Authors:** Mashapa E. Malobane, Adornis D. Nciizah, Patrick Nyambo, Fhatuwani N. Mudau, Isaiah I.C. Wakindiki

**Affiliations:** a Agricultural Research Council - Institute for Soil, Climate and Water, P. Bag X79, Pretoria, South Africa; b University of South Africa, Department of Agriculture and Animal Health, Private Bag X6, Florida, 1710, South Africa; c Department of Agronomy, University of Fort Hare, Private Bag X1314, Alice, 5700, South Africa; d School of Agricultural, Earth and Environmental Sciences, University of Kwazulu Natal, P. Bag X01, Scottsville, 3209, Pietermaritzburg, South Africa

**Keywords:** Conservation agriculture, Soil quality, Soil biological activity, β-Glucosidase, Phosphatase, Urease, Agricultural policy, Agricultural soil science, Agronomy, Organic farming, Fossil fuel

## Abstract

Questions on sustainable and appropriate cropping systems for bioenergy sweet sorghum in the smallholder farming sector still exist. Therefore, a short-term experiment was carried out to study the influence of management on microbial biomass carbon (MBC), β-glucosidase, acid phosphatase, and urease activities in a sweet sorghum cropping system in South Africa. Tillage [no-till (NT) and conventional tillage (CT)], rotation [sorghum-vetch-sorghum (S-V-S) and sorghum-fallow-sorghum (S-F-S)] and residue retention [0%, 15% and 30%] were evaluated. Tillage× rotation× residue management interaction influenced (P < 0.05) MBC whilst crop rotation residue influenced (P < 0.05) β-glucosidase. Tillage affected β-glucosidase (P < 0.05), acid phosphatase (P < 0.001), and urease enzyme (P < 0.01) while crop rotation only influenced acid phosphatase (P < 0.01). Residue retention affected acid phosphatase (P < 0.001) and urease enzyme (P < 0.001). NT + S-V-S+30% interaction resulted in the highest MBC content compared to CT + S-F-S+0%. NT+30% enhanced β-glucosidase activity, S-V-S enhanced acid phosphatase compared to S-F-S. MBC and enzyme activities were positively correlated with each other. Tillage and residue management were the main factors influencing soil biological indicators under bioenergy sweet sorghum in South African marginal soils in the short-term. Soil biological indicators were higher under NT and 30% residue retention respectively. NT + S-V-S+30% was a better treatment combination to enhance soil quality under bioenergy sweet sorghum in South African marginal soils.

## 1 Introduction

Production of sweet sorghum (*Sorghum bicolor* [L.] Moench) for use as biofuel feedstock under smallholder farmer's conditions has the potential to help meet the biofuel targets outlined in the Biofuels Industrial Strategy of the Republic of South Africa [1] at low agronomic inputs. However, uncertainties on sustainable and appropriate sweet sorghum production systems in the smallholder farming sector still exist [2]. Soils in most smallholder farms are degraded [3] with soil organic carbon (SOC) less than 1% [4]. Biofuel feedstock production, which involves harvesting all above-ground biomass, has the potential to worsen soil conditions of these marginal soils [5]. Residue removal increases evaporation and diurnal fluctuations in soil temperature and reduces the input of organic matter needed to improve soil quality [5]. In addition, inappropriate cultivation practices by most smallholder farmers [6] on the already fragile marginal soils exacerbates SOC exhaustion, worsening soil degradation, and food insecurity [4, 7, 8]. Soil degradation negatively influences soil productivity and potential economic returns for smallholder farmers, thus, increasing poverty [9]. Identifying and developing agricultural practices that are suitable for conserving the soil and can result in the sustainable sweet sorghum feedstock production is therefore imperative.

Conservation agriculture (CA) is a potential sustainable production system for sweet sorghum as a biofuel feedstock in South Africa [10], due to its benefits on soil health and crop yields [11]. Conservation agriculture is made up of; minimum soil disturbances, mulch with crop residues, and diversified and economically viable crop rotations [12, 13]. Implementing CA practices enhance SOM build-up and improve soil quality [10, 12, 13, 14] while conserving soil and water [15, 16, 17]. The rate at which SOM is enhanced after the adoption of CA is influenced by site-specific soil conditions, climate, vegetation, residue and rotation management, fertilisation, and other agronomic practices in a given cropping system [18, 19, 20].

Soil organic matter restoration is a slow process [21]. Consequently, it is of interest to study other indicators that are more sensitive to changes in crop and soil management, preferably within the short term after the changes were implemented [22, 23, 24, 25]. Soil microbial properties such as microbial biomass carbon (MBC) and enzyme activities are key indicators of change in soil quality improvement before any significant changes in total soil organic matter can be observed [26, 27, 28, 29, 30]. Soil microbial biomass carbon, which is a measure of the microbial population provides better insights into soil organic C turnover [31]. Soil enzymes play an essential role in catalysing reactions linked with organic matter decomposition and nutrient cycling [24, 32, 33, 34]. Carbon cycling enzymes like β-glucosidase mediate the decomposition of litter and SOM and can help in understanding the effect of management on SOC [28, 35, 36, 37, 38]. Urease enzyme activity is involved in nitrogen cycling whilst phosphatase enzyme activity is involved in phosphorus cycling [28, 36, 39].

Management practices influence MBC and enzyme activities by altering the soil microclimate and soil microorganism habitat, which in turn influences nutrient cycling [40]. Since NT accompanied by residue retention increases organic inputs into the soil and reduces soil disturbance and erosion [29], it generally favours MBC and enzyme activities [29, 41, 42, 43]. Despite the general effect of tillage on MBC and enzyme activities, some authors found tillage not to have any effects on both MBC and enzyme activities [25]. This might be because the type and quality of residues, and rotation, apart from tillage also influenced MBC and enzyme activities [25, 29].

The recent increase in the interest in using marginal soils to increase the production of bioenergy crops has heightened fears of worsening the degradation of the already fragile soils [8, 44, 45, 46]. The impact of CA on the restoration of marginal soils under bioenergy crops in South Africa is currently not known [10]. Nonetheless, evidence of the ability of CA to restore degraded South African soils is currently growing [47]. Much of this evidence is from the studies on physical [48, 49, 50] and chemical [21, 51, 52] soil properties in maize-based cropping systems. However, there is a paucity of information on the effect of management practices and their interactions on biological properties, which are key indicators of change of soil quality in low SOC soils of South Africa. According to Duo et al. [53], while maximising economic returns is at the centre of bioenergy crop production, this does not necessarily equate to maximizing harvested biomass. Appropriate residue retention is important in bioenergy crop production [5, 53]. Nonetheless, more research is still needed to understand the effect of systematic residue retention on soil properties under bioenergy sweet sorghum production in South Africa [10]. The study hypothesised that tillage, crop rotation, and residue management, affect the soil microenvironment, thus, influencing MBC and enzymes activities in low SOC soils. The study aimed to determine the influence of tillage, rotation, and residue management on MBC and activities of β-glucosidase, Urease enzyme, and phosphatase as key indicators of change in soil quality under sweet sorghum cropping system in low SOC soils in South Africa.

## 2 Materials and methods

### 2.1 Study site description

The experiment was conducted at the University of Fort Hare experimental farm which is situated at latitude 32°46ʹ21″S and longitude 26°50ʹ06″E. The experimental site climatic conditions are classified as semi-arid and it receives an annual mean rainfall of about 575 mm during the summer months [54]. The dominant soil form at the experimental site is of alluvial origin, also known as Haplic Cambisol [55]. The soil at the site has 60% sand, 18% silt, 22% clay, pH (H_2_O) 6.98, and SOC 11.5 g kg^−1^ [56].

### 2.2 Experimental design and trial management

Experimental design, treatments, and experimental management were as described by Malobane et al. [57]. The experiment was conducted between October 2016 and March 2019. A randomised complete block designed with a 2 × 2 × 3 split-split-plot arrangement, replicated three times was used in this study. The main plot measured 12.8 m × 17 m and was assigned to tillage treatments (NT and CT). A sub-plot (5.4 m × 17 m) was assigned to crop rotations (S-V-S and S-F-S). Sub-sub-plots (5.4 m × 5 m) were assigned to crop residue management, 0%, 15%, and 30% residue retentions of total fresh harvested biomass. Main plots, sub-plots, and sub-sub-plots were separated by 1 m pathways. Blocks were separated by 2 m pathways. 2.2. Soil sampling.

Soil samples were collected in Mach 2019, after harvesting sweet sorghum. A composite sample made up of three random samples taken at 0.1 m depth in each plot was used for analysis. Noticeable crop residues were removed from the composite sample before analysis. The composite samples were transported to the laboratory on ice. MBC analysis was carried out on the same day of sampling while samples used to determine enzyme activities were passed through a 2 mm sieve after been air-dried.

### 2.3 Analysis

#### 2.3.1 Analysis of soil microbial biomass and enzyme activities

The chloroform fumigation–extraction procedure was used for the determination of MBC following the methods of Anderson and Ingram [58]. K_c_ of 0.38 was used [59].

Urease enzyme activity was measured using the colorimetric method outlined by [60] after incubating 5 g air-dried soil samples with a urea solution for 2 h at 37 °C.

β-Glucosidase enzyme activity was measured using the colorimetric method described by [61] after incubating 1 g air-dried soil sample with p-Nitrophenyl-β-D-glucoside and modified universal buffer solution (pH 6.0) for 1 h at 37 °C.

Acid phosphatase activity was measured using the colorimetric method as described by [61] after incubating 1 g air-dried soil sample with toluene, modified universal buffer (pH 6.5), and p-nitrophenyl phosphate for 1 h at 37 °C.

#### 2.3.2 Statistical analysis

The JMP 14.0 version was used to perform a three-way analysis of variance (ANOVA). The least significant difference method at *P* ≤ 0.05 was used for mean separations.

## 3 Results

### 3.1 Microbial biomass carbon

**Figure 1:**
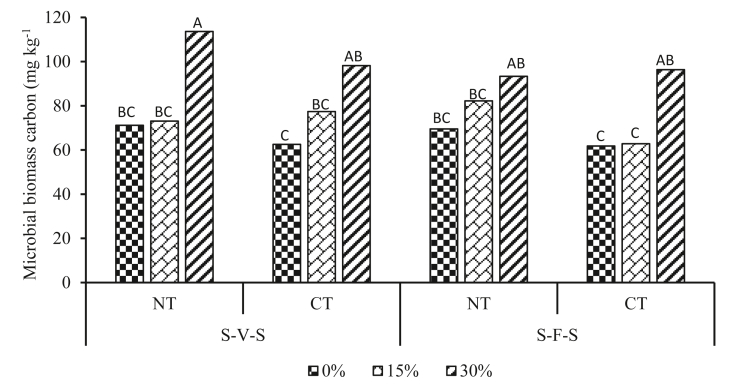
Tillage, crop rotation, and residue management interaction influence on MBC. Different uppercase letters indicate significant differences at P ≤ 0.05. NT: no-till, CT: conventional tillage, S-V-S: sweet sorghum-grazing vetch-sweet sorghum, S-F-S: sweet sorghum-fallow-sweet sorghum, bars with similar letters are not statistically different.

Tillage × crop rotation × residue management interaction influenced (P < 0.05) MBC (Table 1). Retention of 30% residues increased MBC regardless of tillage or rotation (Figure 1). Microbial biomass carbon was 84% higher in NT + S-V-S + 30% than in CT + S-F-S + 0% treatments. However, there were no differences between 0% and 15% residue retention both tillage and rotation levels.

**Table 1:** Analysis of variance (ANOVA) for microbial biomass carbon (MBC), β-glucosidase, acid phosphatase, and urease enzyme activity as affected by tillage, crop rotation, crop residue management, and their interaction.

	MBC	β-Glucosidase	Acid phosphatase	Urease
Tillage (T)	∗	∗	∗∗∗	∗∗
Rotation (CR)	ns	ns	∗∗	ns
Residue. management(R)	∗∗∗	∗∗	∗∗∗	∗∗∗
T × CR.	ns	ns	Ns	ns
T × R	ns	ns	Ns	ns
CR × R	ns	∗	Ns	ns
T×CR × R	∗	ns	Ns	ns

ns: not significant, ∗, ∗∗, ∗∗∗ significant difference at 0.05, 0.01 and 0.001, probability level, respectively.

### 3.2 β-Glucosidase activity

**Figure 2:**
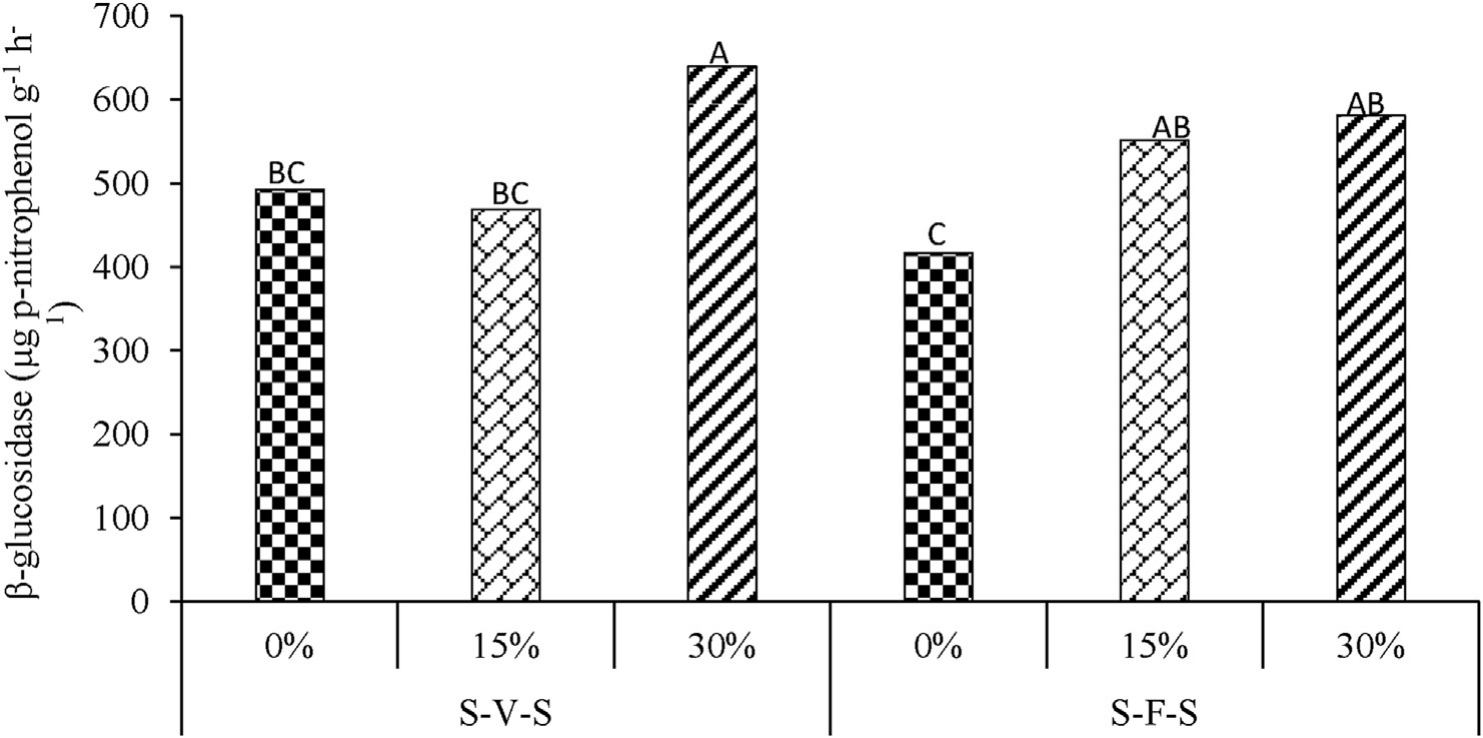
Rotation × residue management interaction effects on β-glucosidase activity. Different uppercase letters indicate significant differences at P ≤ 0.05. S-V-S: sweet sorghum-grazing vetch-sweet sorghum, S-F-S: sweet sorghum-fallow- sweet sorghum.

**Figure 3:**
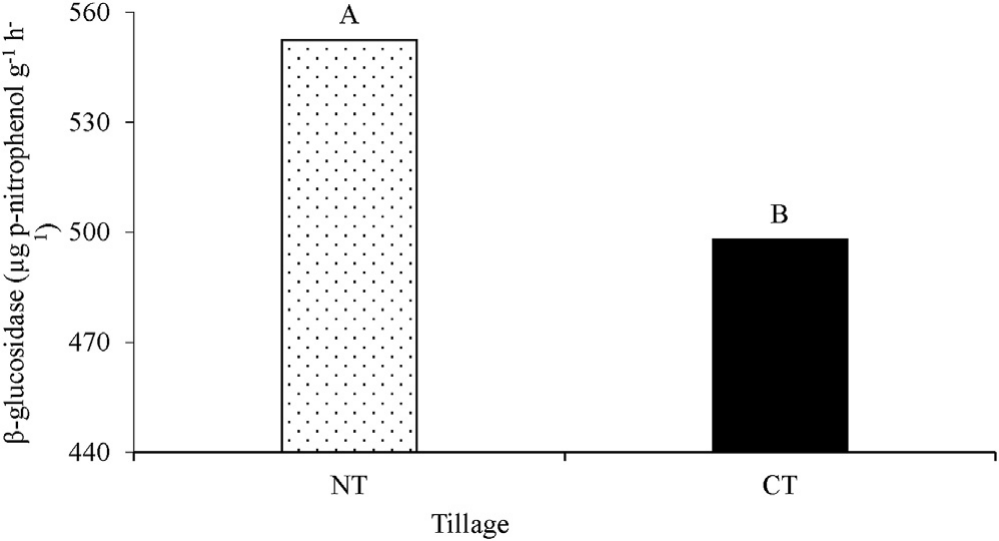
Tillage effects on β-glucosidase activity. Different uppercase letters indicate significant differences at P ≤ 0.05. NT: no-till and CT: conventional tillage.

No significant three-way interactions of tillage x crop rotation x crop residue management were observed (P > 0.05) concerning β-Glucosidase activity (Table 1). The two-way interaction of crop rotation and residue management (P < 0.05) was significant. The main effects of tillage (P < 0.05), and residue management (P < 0.01) affected β-Glucosidase activity. The S-V-S rotation + 30% residue retention resulted in the highest β-glucosidase activity followed by S-F-S + 30% residue retention while S-F-S + 0% residue retention had the lowest (Figure 2). The activity ranged from 640.38 in S-V-S + 30% to 417,11 μg p-nitrophenol g^−1^ h^−1^ in S-F-S + 0% residue management practice. The β-glucosidase activity was significantly higher under NT compared to CT treatment (Figure 3). Under, S-V-S rotation, retention of 30% residues resulted in the highest β-glucosidase activity, whilst no differences were observed between 0 and 15%. Conversely, under S-F-S, there were no differences in activity between 15 and 30% residue retention, even though 0% had the lowest.

### 3.3 Acid phosphatase activity

Tillage × crop rotation × crop residue management interaction was not significant (P > 0.05) concerning acid phosphatase activity (Table 1). The main effects of tillage (P < 0.001), crop rotation (P < 0.01), and residue management (P < 0.0001) significantly affected acid phosphatase activity. The implementation of NT resulted in a 21% greater acid phosphatase activity than in the CT treatment (Table 2). S-V-S treatment resulted in an 8% greater acid phosphatase activity than S-F-S (Table 2). Acid phosphatase activity was 11% and 25% greater in 30% residue retention than in 15% and 0% residue retention management, respectively. Acid phosphatase activity was 13% greater in 15% residue retention than in 0% residue retention management.

**Table 2:** Tillage, rotation, and residue management effects on acid phosphatase and β-glucosidase activity.

Treatment	Acid phosphatase activity (μg p-nitrophenol g^−1^ h^−1^)	Urease activity (μg NH_4_-N g^−1^ 2h^−1^)
**Tillage**		
no-till	2131.18a	24.10a
conventional tillage	1766.65b	20.77b
	P value_< 0.001_	P value_< 0.01_
**Rotation**		
sorghum-vetch-sorghum	2026.94a	23.41a
sorghum-fallow-sorghum	1870.88b	21.47a
	P value_< 0.01_	
**Residue management**		
0%	1733.01c	24.10a
15%	1952.14b	20.77b
30%	2161.59a	24.10a
P value < 0.001		

Numbers followed by different letters in the same row indicate differences among the treatments.

### 3.4 Urease activity

Tillage × crop rotation × crop residue management interaction was not significant (P > 0.05) concerning urease activity (Table 1). The activity of urease was affected by tillage (P < 0.01) and residue management practices (P < 0.001) (Table 1). Urease activity was 16% greater under NT compared to CT treatment (Table 2). Retention of 30% residues increased urease activity compared to 15% and 0% residue management (Table 2). Urease activity under 30% residue retention was 40% and 47% greater than in 15% and 0% residue retention management, respectively.

### 3.5 Correlations between MBC, β-glucosidase, acid phosphatase, and urease activity

MBC was strongly positively correlated to selected enzyme activities (Table 3). In addition, the activities of the selected enzymes were strongly positively correlated to each other.

**Table 3:** Correlations between microbial biomass carbon (MBC), β-Glucosidase, acid phosphatase, and urease activity.

	MBC	β-Glucosidase	Acid phosphatase	Urease
MBC	1			
β-Glucosidase	0.75	1		
Acid phosphatase	0.79	0.76	1	
Urease	0.94	0.78	0.83	1

## 4 Discussion

Soil is recognized as a non-renewable resource that is vital for food security and a sustainable human future [14, 62]. Thus, the soil requires continuous monitoring to avoid its degradation and enhance its sustainability [62, 63]. For this reason, biological indicators are identified as sensitive soil quality indicators for early change in soil quality after the adoption of new soil management [64, 65, 66]. The influence of tillage, crop rotation, crop residue management, and their interaction on MBC, β-glucosidase, acid phosphatase, and urease enzyme activity were studied.

In this study, NT + SVS + 30%, which adheres to the three principles of CA increased MBC more than the rest of the treatment combinations. The application of NT minimises the disturbance of soil microbial life and enhances soil organic carbon compared to CT treatment [67], which in turn promotes MBC production [68]. Residues provide food for microbial growth and multiplication [69] hence higher levels of MBC in 30% residue retention than other residue management practices. In addition, the crop in rotation provides an additional substrate for microbial life and multiplication [70, 71]. The increase in MBC after implementation of NT + SVS + 30%, is a sign of soil quality restoration [72, 73], which enhances the soil's capacity to carry out ecosystem processes [74] and is also positively related to nutrients availability [75]. The findings from this study also suggest that MBC is less sensitive to change in crop residue retention amount under bioenergy sweet sorghum production system in South African marginal soils. As can be observed in Figure 1, a meaningful change in MBC requires a higher amount of residue retention under both tillage levels plus both crop rotations. However, the trend was statically not clear under CT + S-V-S+ residue retention and NT + S-F-S + residue retention. The reason for such an unclear trend is currently unknown.

This study supports previous findings by [76], who reported that rotation had less impact on most enzyme activities in a short-term study. However, findings from this study contradict findings by Muzangwa et al. [30], Njaimwe et al. [49], and Mukumbareza et al. [77] who found crop rotation to increase enzyme activities under low SOC in a short-term study. The inclusion of grazing vetch in crop rotation was found to increase enzyme activities compared to the system with winter fallow [77]. The minimal influence of rotation on measured variables might be due to low grazing vetch biomass in winter due to low rainfalls during the study. The application of NT and 30% residue retention in marginal soils, had the highest amount of β-glucosidase activity. This supports previous work that found NT and residue retention to be critical in enhancing β-glucosidase activity under soils with low SOC [30, 40, 77, 78]. The study by Mukumbareza et al. [77] and Muzangwa et al. [30] in the maize production system in South African under the similar soil conditions of the current study, included 100% maize stalks retention to observe an increase in β-glucosidase activity. Thus, finding from this study suggest that an increase in β-glucosidase activity can be observed even at a minimal of 30% residue retention in marginal soils of South Africa under bioenergy sweet sorghum production. The residues applied in the NT treatment decompose slowly leading to the accumulation of soil organic matter on the surface, which stimulates biological activities and the resulting increase in enzyme activities [28, 68, 79, 80]. β-Glucosidase activity is important in carbon-cycling as it is involved in catalysing the hydrolysis of cellobiose that yields a vital energy source for soil biological activities [28, 35, 81]. The lower β-glucosidase activity under CT treatment and residue removal treatments suggest the application of NT and residue treatments have the ability to increase soil microbial properties, and possibly impact C dynamics and soil fertility [28, 35].

To enhance soil productivity and quality, it is vital to assess the impact of different management practices on soil phosphatase activity [28]. The increase in acid phosphates under NT [82, 83, 84, 85], rotation [30, 77, 86, 87] and residue retention [30, 84, 88, 89] were previously reported even under soils with low SOC. In this study, the application of NT, S-V-S, and 30% increased acid phosphatase activity compared to CT, S-F-S, and residue removal, respectively. The increase in acid phosphatase is mainly due to the increase in microbial growth and soil organic matter enrichment [28, 85] under NT, rotation, and residue retention compared to CT, monoculture, and residue removal. The increase in phosphatase leads to an increase in phosphorus availability and enhanced soil fertility [28, 77].

Urease activity analysis can tell management practices that best enhance microbial metabolism and nitrogen cycling [28]. Eivazi et al. [26], Adetunji et al. [28], Roldán et. [90], and Raiesi and Kabiri [91], found an increase in urease activity in NT compared to CT, which supports the findings in this study. The increase in urease activity under 30% residue retention in this study, support previous findings by Ji et al. [92], Zhang et al. [93], and Saikia and Sharma [94], who found residue retention to be crucial in improving urease activity. Increased urease activities might be due to improved soil organic carbon, organic nitrogen, and biological activity [28] after NT and residue retention. The increase in MBC and soil enzyme activities in this study demonstrate restoration in soil quality, which has a potential to unlocked sustainability and economical success of marginal soils [95].

Correlations between MBC and soil enzyme activities may provide knowledge about how the biological population influences enzyme activities, which are responsible for nutrients cycling. The positive correlations between MBC and soil enzyme activities might be due to the increase in soil organic carbon after the implication of NT, crop rotation, and residue management, which stimulate microbial population measured as MBC, which then enhances enzyme activities [28, 96].

## 5 Conclusions

Tillage and residue management were critical in increasing MBC, β-glucosidase, acid phosphatase, and urease activity in the short-term under poor soils of South Africa. No-till and 30% residue retention significantly enhanced MBC and selected enzyme activities compared to CT and residue removal treatment, respectively. The response of MBC and selected enzyme activities under NT and 30% emphasizes the importance of these management practices in conserving soil health. The increase in MBC under NT + S-V-S + 30% treatment combination, suggesting that it has the potential to serve as a CA practice to enhance soil quality under bioenergy sweet sorghum cropping system in low organic C soils in South Africa. Future studies should include the use of summer crop rotation and intercropping to maximize the potential benefits of crop rotation in South African marginal soils under rainfed conditions.

## Declarations

### Author contribution statement

Mashapa E. Malobane; Adornis D. Nciizah; Patrick Nyambo: Conceived and designed the experiments; Performed the experiments; Analyzed and interpreted the data; Contributed reagents, materials, analysis tools or data; Wrote the paper.

Fhatuwani N. Mudau; Isaiah I. C. Wakindiki: Analyzed and interpreted the data; Wrote the paper.

### Funding statement

This work was supported by 10.13039/501100001321National Research Foundation of South Africa (grant number 98690).

### Data availability statement

Data included in article.

### Declaration of interests statement

The authors declare no conflict of interest.

### Additional information

No additional information is available for this paper.
